# Identifying the shared genes and their related microRNAs, metabolites, and pathways in ischemic stroke and epilepsy

**DOI:** 10.1038/s41598-026-39299-5

**Published:** 2026-02-10

**Authors:** Yu Chen, Shuhong Man, Qinfeng Li, Yuelong Ji, Biwen Peng, Yansheng Ding, Jian Xu

**Affiliations:** 1 Department of Clinical Laboratory, Weifang Maternal and Child Health Hospital, No. 12007, Yingqian Street, High-tech Zone, Weifang, 261011 Shandong China; 2 Department of Obstetrics and Gynecology, Weifang People’s Hospital, Weifang, 261000 Shandong China; 3 School of Public Health, Peking University, Beijing, 100191 China; 4 Department of Physiology, School of Basic Medical Sciences, Wuhan University, Wuhan, 430071 China; 5 Wuhan University School of Basic Medical Sciences-Weifang Children’s Neurological Diseases and Innovation Transformation Joint Research Center, Weifang, 261011 Shandong China

**Keywords:** Ischemic stroke, Epilepsy, Shared genes, MicroRNA, Metabolites, Biomarkers, Computational biology and bioinformatics, Diseases, Genetics, Neurology, Neuroscience

## Abstract

Background This study aimed to identify shared genes between ischemic stroke (IS) and epilepsy and explore underlying mechanisms. Methods Transcriptomic datasets from the GEO database were analyzed using differential expression and weighted gene co-expression network analysis (WGCNA). Hub-shared genes were identified through protein-protein interaction networks, ROC analysis, and expression validation. Upstream miRNAs were predicted. Additionally, untargeted plasma metabolomics was performed on children with epilepsy and healthy controls, followed by differential metabolite analysis and metabolic pathway construction. Results WGCNA revealed 594 epilepsy-related and 2,623 IS-related DEGs, with 38 shared DEGs identified, including IL10RA, CD2, and C3AR1. These genes showed high diagnostic value, with their AUC value > 0.66 in both training and validation datasets. Additionally, hsa-let-7b-5p was predicted to target C3AR1. Metabolomics identified 139 differential metabolites, and C3AR1 was implicated in synaptic vesicle cycle, taste transduction, and nicotine addiction pathways via acetylcholine. Conclusions The shared genes, especially C3AR1 may be a key regulator in the development IS and epilepsy, showing potential as a biomarker for both diseases. However, its diagnostic efficacy requires further clinical validation. Given the complexity of these diseases, future research may focus on identifying a panel of biomarkers rather than relying on a single gene.

**Supplementary Information:**

The online version contains supplementary material available at 10.1038/s41598-026-39299-5.

## Introduction

 Stroke is a major cause of disability worldwide and seriously affects living standards and quality of life. There are two types of stroke: ischemic stroke (IS) and hemorrhagic stroke. IS is the most common type of stroke and accounts for approximately 80% of all strokes^1^. Cardioembolic stroke is a subtype in which cerebral thromboembolic ischemia is caused by cardiogenic emboli, which accounts for 14%–30% of IS and is more likely to cause disability^2^. Stroke is recognized as one of the most common causes of epilepsy, with post-stroke epilepsy (PSE) accounting for 11% of all epilepsies in the adult population^3,4^. Epilepsy is a common neurological disorder characterized by repeated seizures. It affects approximately 65 million people worldwide and imposes a great burden on individuals and health systems^5,6^. To date, managing PSE does not completely match the treatment of stroke and epilepsy caused by other reasons, and neurologists lack guidance on managing PSE, such as when and how to treat patients. Therefore, investigating the pathogenesis and association between stroke and epilepsy is necessary.

The risk of seizures is high in many post-stroke patients, and the incidence of PSE has been increasing over the last decades^7,8^. Although its incidence after hemorrhagic stroke is higher than that after IS, the overall seizure burden in the IS group is greater because of the higher frequency of IS^9^. Common pathophysiological mechanisms are assumed to underlie stroke and epilepsy^10^. Following a stroke, various inflammatory mediators, such as prostaglandins, cytokines, chemokines, damage-associated molecular patterns (DAMPs), and complements are released to repair brain damage. When sustained over time, neuroinflammation causes neuronal and astroglial dysfunctions, resulting in altered synaptic transmission, neuronal loss, hyperexcitability, and abnormal neurogenesis^11^. These mechanisms contribute to epilepsy development^12–14^. Moreover, some less common factors that may cause seizures, such as the central nervous system (CNS) infections, systemic metabolic abnormalities, such as hypocalcemia and hypo/hyperglycemia, and benzodiazepines and barbiturates, may also coexist in patients with strokes, suggesting a similar etiology of seizures and strokes^15^. Furthermore, early PSE is associated with high levels of the neural cell adhesion molecule and low levels of tumor necrosis factor-R1^16^. A recent study has identified hub genes such as TPGS2, TMCC3, GADD45B, and KCNJ2 as key players in both IS and epilepsy, suggesting that these genes may play a key role in the co-pathogenesis of IS and epilepsy^17^. Although a strong association exists between stroke and epilepsy from the clinical and epidemiological perspectives, the shared genetic and metabolic mechanisms in IS and epilepsy are largely unknown.

In this study, we downloaded the IS- and epilepsy-related datasets from the Gene Expression Omnibus (GEO) database and analyzed the shared genes of the two diseases using differential expression analysis and weighted gene co-expression network analysis (WGCNA). We identified hub-shared genes via protein-protein interaction (PPI) analysis, receiver-operating characteristic (ROC) curve analysis, and differential expression validation using validation datasets. Moreover, we predicted the miRNAs upstream of the hub-shared genes. Furthermore, we performed nontargeted metabolomics analysis of the plasma samples from children with epilepsy and healthy controls, analyzed key differential metabolites, and constructed a metabolic pathway network of the hub-shared genes. A flowchart of the study is shown in Supplementary Fig. 1. Our study aimed to explore the shared genetic and metabolic mechanisms between IS and epilepsy, offering new insights for identifying potential targets that could be useful in treating PSE.

## Methods

### Data acquisition and preprocessing

The IS-related gene expression profiles GSE58294 (training dataset) and GSE16561 (validation dataset), as well as the epilepsy-related gene expression profiles GSE143272 (training dataset), GSE16969 (validation dataset) and GSE205661 (validation dataset), were downloaded from the NCBI GEO database^18^. The detailed information of these datasets is listed in Table 1.

**Table 1:** The detail information about datasets.

Disease	Datasets		Disease samples	Control samples	Platform
IS	GSE58294	Training	21 cardioembolic stroke (time after stroke: 3 h)	20 control	GPL570 [HG-U133_Plus_2] Affymetrix Human Genome U133 Plus 2.0 Array
	GSE16561	Validation	39 IS	24 control	GPL6883 Illumina HumanRef-8 v3.0 expression bead chip
Epilepsy	GSE143272	Training	12 epilepsy (4 idiopathic, 4 symptomatic and 4 cryptogenic epilepsy)	4 control	GPL570 [HG-U133_Plus_2] Affymetrix Human Genome U133 Plus 2.0 Array
	GSE16969	Validation	4 epilepsy	4 control	GPL570 [HG-U133_Plus_2] Affymetrix Human Genome U133 Plus 2.0 Array
	GSE205661	Validation	6 temporal lobe epilepsy with hippocampal sclerosis (TLE + HS)	9 control	GPL19072 Agilent-052909 CBC_lncRNAmRNA_V3 (Probe Name version)

The mRNA probe expression matrix of each dataset and the annotation file of the detection platform were downloaded. Probes were individually converted into gene symbols. Probes that did not match the gene symbols were removed, and the average expression of multiple probes mapped to the same gene symbol was selected.

### Differential expression analysis

Based on the training datasets (GSE143272 epilepsy dataset and GSE58294 IS dataset), the differentially expressed genes (DEGs) in the epilepsy and IS samples were compared with their respective control samples using the R limma package (version 3.48.3)^19^. The P value was adjusted by Benjamini-Hochberg method. The adjusted P (P.adj) value < 0.05 and |log fold change (FC)| > 0.263 (that was FC > 1.20 for upregulation and FC < 0.8 for downregulation)^20^ were set as the cutoff values for DEG screening.

### Identifying disease-related genes using WGCNA

The disease-related key modules and module genes were selected by conducting WGCNA on the DEGs identified from peripheral blood samples in the training datasets of two diseases to construct gene co-expression modules using the R WGCNA package (version 1.71)^21^. Briefly, an appropriate soft-thresholding power was selected to obtain a scale-free network. The Pearson correlation coefficient between the two diseases is determined through the use of formula 1:

$$\:{\mathrm{S}}_{\mathrm{i}\mathrm{j}}=\left|\frac{1+\mathrm{c}\mathrm{o}\mathrm{r}\left({\mathrm{x}}_{\mathrm{i}}+{\mathrm{y}}_{\mathrm{i}}\right)}{2}\right|,$$

where *i* and *j* are the expression levels of the *i*-th gene and the *j*-th gene respectively.

Then, formula 2 and 3 are used to convert the gene expression similarity matrix into an adjacency matrix, with the network type being signed, where *β* is the soft threshold, which is the square of the Pearson correlation coefficient *β* for each pair of genes. The adjacency matrix is converted into a topology matrix, and the topology overlap (Topological overlap measure, TOM) is used to describe the degree of association between genes:

$$\:a_{{ij}} = \left| {\frac{{1 + cor\left( {x_{i} + y_{i} } \right)}}{2}} \right|^{{\beta \:}} \;{\mathrm{and}}\;\: = \frac{{\sum _{{u \ne ij}} a_{{iu}} a_{{uj}} + a_{{ij}} }}{{min\left( {\sum _{u} a_{{iu}} + \sum _{u} a_{{ju}} } \right) + 1 - a_{{ij}} }}$$

Gene co-expression modules were determined, and genes with similar expression patterns were clustered.Using formula 4 for clustering analysis, 1-TOM represents the degree of difference between genes *i* and *j*. Genes are hierarchically clustered, followed by module identification using dynamic cutting tree methods. The most representative gene in each module is called a feature vector gene, or ME for short. It represents the overall level of gene expression within the module and is the first principal component in each module. Here, *i* denotes the gene in module *q*, and *l* denotes the chip sample in module *q*:

$$\:ME=princomp\left({x}_{il}^{\left(q\right)}\right).$$

Module Identity (MM) is used to measure the association degree of a gene with a specific module. This is achieved by calculating the Pearson correlation coefficient between the expression profile of a gene in all samples and the eigenvector gene $\:{\mathrm{M}\mathrm{E}}^{\mathrm{q}}$ of module *q*. This process can be completed using formula 5: $\:{\mathrm{M}\mathrm{M}}_{\mathrm{i}}^{\mathrm{q}}=\mathrm{c}\mathrm{o}\mathrm{r}\left({\mathrm{x}}_{\mathrm{i}},{\mathrm{M}\mathrm{E}}^{\mathrm{q}}\right)$. Here, $\:{\mathrm{x}}_{\mathrm{i}}$represents the expression profile of the *i*-th gene, $\:{\mathrm{M}\mathrm{E}}^{\mathrm{q}}$ is the eigenvector gene expression profile of module *q*, and $\:{\mathrm{M}\mathrm{M}}_{\mathrm{i}}^{\mathrm{q}}$ is the Module Identity value of gene *i* in module *q*. When $\:{\mathrm{M}\mathrm{M}}_{\mathrm{i}}^{\mathrm{q}}$ equals 0, it indicates that the gene does not belong to module *q*; if the value of $\:{\mathrm{M}\mathrm{M}}_{\mathrm{i}}^{\mathrm{q}}$approaches + 1 or −1, it suggests that gene *i* has a stronger correlation with module *q*. A positive sign indicates a positive correlation between gene *i* and module *q*, while a negative sign indicates a negative correlation.

Finally, it is analyzed and visualized. The cluster tree was cut into branches to define the modules using a dynamic tree cut, and each module was assigned a different color for visualization. Each module contained at least 30 genes. The epilepsy- or IS-related key modules were identified using hierarchical clustering and correlation analysis of the module eigengene values and clinical traits. Disease-related DEGs were identified from key epilepsy- and IS-related modules.

### Analyzing the shared genes of epilepsy and IS

The overlapping DEGs in the epilepsy- and IS-related key modules were considered as the shared genes of two diseases and were obtained using Venn diagram analysis using the R VennDetail package (version 1.2.0).

### Functional enrichment analysis for shared genes

The shared genes of two diseases were imported for Gene Ontology (GO) functions and Kyoto Encyclopedia of Genes and Genomes (KEGG)^22–24^ pathway enrichment analyses using the R clusterProfiler package (version 4.44)^25^. GO describes the overrepresented biological functions from three aspects: molecular function (MF), cellular component (CC), and biological processes (BP).

### PPI network analysis and hub gene identification

The PPI relationships of the shared genes of two diseases were analyzed using the STRING (version 11.0) database^26^, followed by the construction of a PPI network using Cytoscape (version 3.9.2)^27^. The interaction score was set to 0.15. The top five hub genes were analyzed using the cytoHubba plug-in^28^, and four topological analysis algorithms, including the maximal neighborhood component (MNC), maximal clique centrality (MCC), degree, and edge-percolated component (EPC). MCC calculated the importance of nodes by identifying the maximal cliques in the network MNC evaluated node importance based on the strength of interactions with neighboring nodes. Degree assessed node importance by calculating its number of connections. EPC identified key edges and nodes in the network through edge percolation. These algorithms were widely utilized for selecting hub genes from PPI networks^29,30^. The intersecting genes of the top five genes from each topological analysis algorithm were considered candidate hub genes for the two diseases.

### Diagnostic efficacy evaluation of the hub-shared genes

To evaluate the diagnostic efficacy of the candidate hub-shared gene, ROC curves were plotted using the R pROC package (version 1.18.0)^31^ based on the expression data of the hub-shared genes in all the enrolled datasets. The higher the value of the area under the curve (AUC), the stronger the diagnostic efficacy was.

### Validating the differential expression of the candidate hub-shared genes

Using the validation datasets of IS (GSE16561) and epilepsy (GSE16969 and GSE205661), the differential expression of the candidate hub-shared genes in the two diseases was validated. Differential expression analysis of the candidate hub-shared genes was performed using Student’s t-test. Genes with the same expression trends in the disease samples in the training and validation datasets were considered the hub-shared genes.

### PPI network of the hub-shared genes

The possible mechanisms of the hub-shared genes were further studied by performing its PPI analysis with 20 interacting genes using the GeneMANIA database^32^. Thus, their co-localization, shared protein domains, co-expression, and pathways were explored.

### Prediction of the upstream MiRNAs of the hub-shared genes

The common miRNAs associated with the two diseases were identified using the Human microRNA Disease Database (HMDD)^33^. Moreover, miRNAs upstream of hub-shared genes were predicted using the miRWalk database, and an miRNA-gene network was established. Disease-related miRNAs of the hub-shared genes were identified using the intersection analysis of the disease-related miRNAs and upstream miRNAs of the hub-shared genes.

### Collection of clinical samples for nontargeted metabolomics analysis

Fourteen plasma samples from children with epilepsy and 20 plasma samples from healthy controls were collected from Weifang Maternal and Child Health Hospital. All patients with epilepsy met the diagnostic criteria for epilepsy. Exclusion criteria included: (1) other neurological diseases or genetic disorders; (2) somatic or psychiatric diseases that could potentially affect metabolism; (3) factors such as infections or immune deficiencies that significantly impact metabolism. All children in this study had not used any antiepileptic drugs, central nervous system medications, or immunosuppressants during the treatment process. The study was approved by Weifang Maternal and Child Health Hospital, and the guardians of children signed written informed consent.

### Nontargeted metabolomics analysis and differential metabolite identification

Metabolomics analyses were conducted using ultra-high performance liquid chromatography (1290 Infinity LC, Agilent Technologies) coupled to a quadrupole time-of-flight (AB Sciex TripleTOF 6600) at Beijing Allwegene Technology Co., Ltd. (Beijing, China). In detail, the samples were added to cold methanol/acetonitrile/H_2_O (2:2:1) solution, shaken and extracted for 30 min. Then, the samples were centrifuged at 14,000 g at 4℃ for 20 min, and the supernatant were subjected to vacuum drying. The residue was dissolved in 100 µL acetonitrile solution and centrifuged at 14,000 g at 4 °C for 10 min, and the supernatant was collected for liquid chromatography–mass spectrometry (LC–MS) analysis. The LC conditions were as follows: the Vanquish ultrahigh-performance liquid chromatograph (UHPLC) (Thermo) with Waters, ACQUITY UPLC BEH Amide 1.7 μm, 2.1 mm × 100 mm column was used, with the column temperature of 25 °C, flow rate of 0.3 mL/min, and sample size of 2 µL. Mobile phase A consisted of H_2_O, 25 mM ammonium acetate, and 25 mM ammonia water. Mobile phase B was acetonitrile. The solvent gradient was set as follows: 98% B, 1.5 min; 98–2% B, 12 min; 2% B, 14 min; 2–98% B, 14.1 min; 98% B, 17 min. The samples were placed in a 4 °C automatic injector during the entire analysis process. The quality control (QC) samples were inserted into the sample queue to monitor and evaluate the stability of the system and the reliability of the experimental data. Using the QExactive mass spectrometer, the primary and secondary spectra of samples were collected. This mass spectrometer provided electrospray ionization (ESI) positive and negative ion detection modes. ESI source conditions were as follows: the ion source gas 1: 60, ion source gas 2: 60, curtain gas (CUR): 30 psi, source temperature: 600 °C; ion spray voltage floating (ISVF): ± 5500 V, primary mass-charge ratio detection range: 80–1200 Da, resolution: 60,000, accumulation time: 100 ms, scanning range of secondary stage: 70–1200 Da, resolution: 30,000, scan accumulation time: 50 ms, and dynamic exclusion time: 4 s. The raw data were subjected to peak alignment, retention time correction and peak area extraction using XCMS software. Multivariate statistical analyses were performed. Subsequently, the differential metabolites were identified with the cutoff values of *p* < 0.05 (Student’s t-test), FC > 1.5 or < 0.67^34,35^, and variable importance in the projection (the first principal component of orthogonal partial least squares discriminant analysis) > 1.

### Pathway enrichment analysis for the hub-shared genes and differential metabolites

To clarify the biological significance of the hub-shared genes and differential metabolites, the pathways significantly enriched by the hub-shared genes and differential metabolites were analyzed using the IMPaLA^36^ tool with the background of the KEGG database. The screening parameters were set as follows: pathway_source, KEGG; num_overlapping_metabolites/genes > 0; and p_joint < 0.05.

### Metabolic pathway enrichment analysis

To further understand the overall affected pathways and corresponding differential metabolites, metabolic pathway enrichment analysis was performed to map a metabolic pathway network using FELLA, an R package to perform a network-based enrichment of a list of affected metabolites^37^. Enrichment was calculated based on the KEGG pathways using the diffusion method. According to the results of the pathway enrichment analysis, with the KEGG graph as a large background, a metabolite pathway background network was constructed, containing pathways, metabolites, reactions, enzymes, and modules. Using the diffusion algorithm, heat was forced to flow from a given node (the metabolites in the significantly enriched pathway) and pass through the metabolite pathway background network, leading to the changes in the score of each node in the background network until the final rest. The temperatures (diffusion scores) were calculated as follows:

$${\text{T }} = {\text{ }} - {\mathrm{KI}} - {\text{1 }} \times {\text{ G}}.$$

G is the heat generation vector (1 is the differential metabolite and 0 otherwise); KI is the conductance matrix, where KI = L + B, L is the unnormalized graph Laplacian, B is the diagonal adjacency matrix with Bii = 1 if node i is a pathway, and Bii = 0 otherwise. Matrix B ensures that the flow leaves the graph through the nodes of the pathways.

The p-value was obtained using a permutation test according to the diffusion scores. The network nodes were sorted according to the p-value, and nodes with p-value < 0.05 were screened to construct the metabolic pathway network.

## Results

### Identification of DEGs in epilepsy and IS

**Fig. 1:**
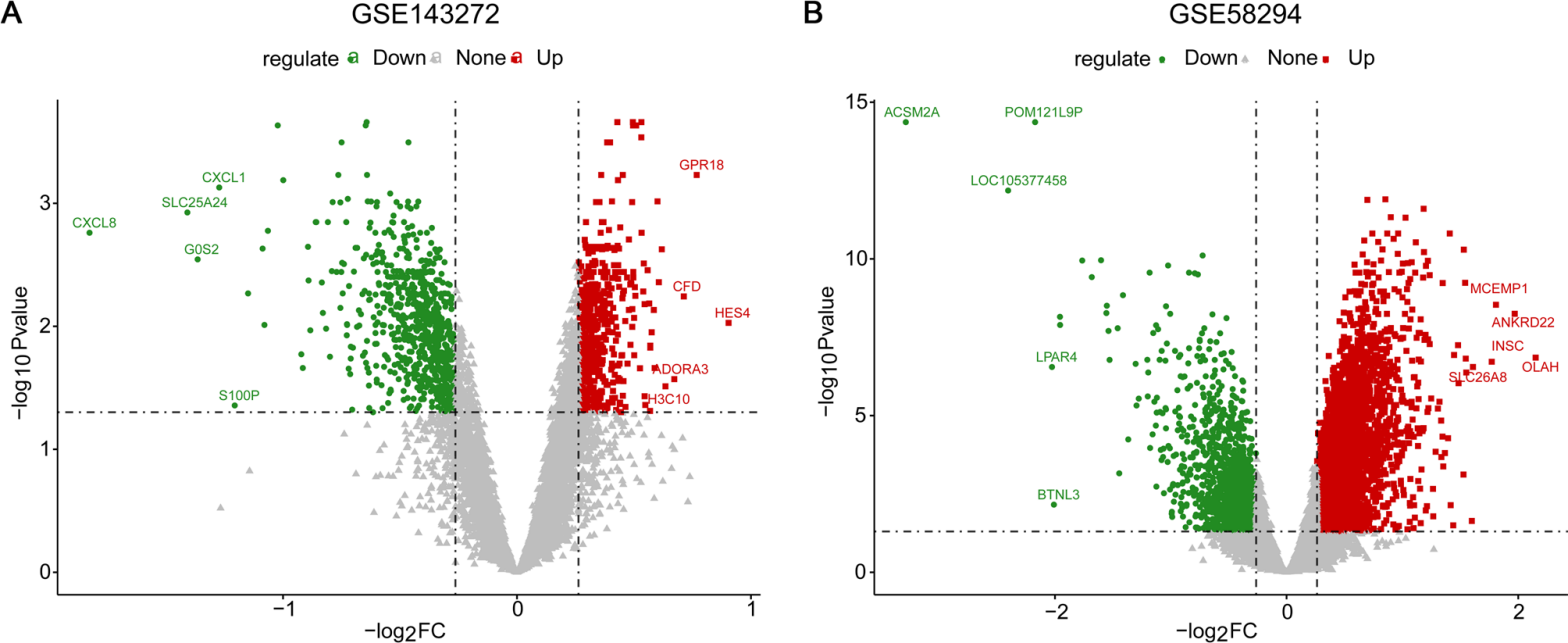
Volcano plots of DEGs associated with epilepsy and IS. **A** Volcano plot of DEGs between epilepsy and control samples based on the GSE143272 dataset. **B** Volcano plot of DEGs between IS and control samples based on the GSE58294 dataset. The horizontal axis is −log10 (pValue), and the vertical axis indicates log2 (FC). Each node represents a gene. The red nodes indicate upregulated genes, the green nodes indicate downregulated genes and the gray nodes indicate genes without significant differences. IS: ischemic stroke; DEGs: differentially expressed genes; FC: fold change.

We performed a differential expression analysis to identify DEGs in epilepsy and IS. A total of 549 upregulated and 739 downregulated DEGs between the epilepsy and control samples were identified based on the GSE143272 epilepsy dataset (Supplementary Table 1), and the volcano plot of these DEGs is shown in Fig. 1A. In addition, 3593 upregulated and 1410 downregulated DEGs between the IS and control samples were screened based on the GSE58294 IS dataset (Supplementary Table 2). The volcano plot of these DEGs is shown in Fig. 1B.

### Analysis of the co-expression module and identification of the disease-related genes

**Fig. 2:**
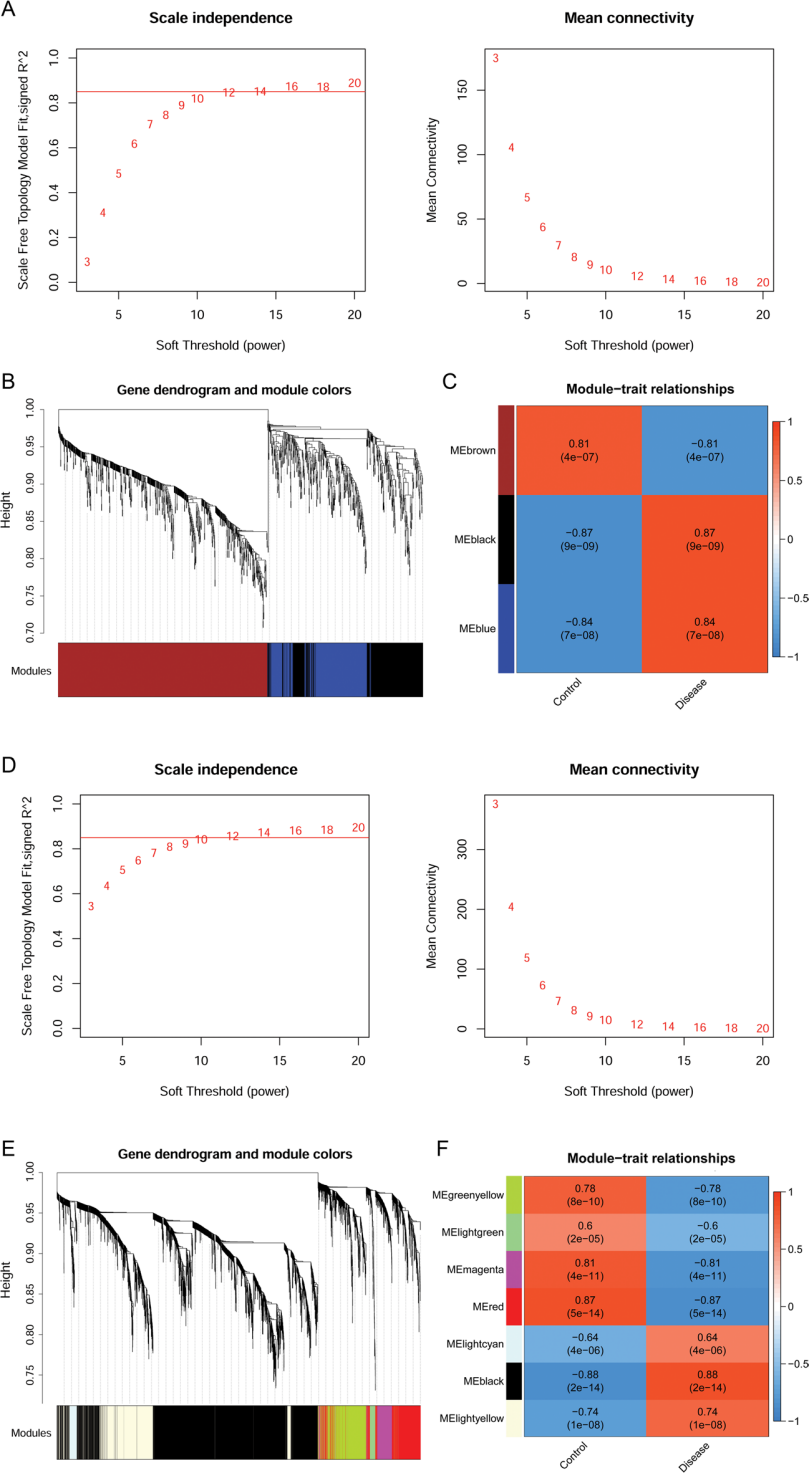
Analyzing epilepsy- and IS-related gene coexpression modules using WGCNA based on GSE143272 and GSE58294 datasets, respectively. **A** Analysis of scale independence and mean connectivity to determine soft-thresholding power in WGCNA based on the GSE143272 dataset. **B** Clustering dendrograms of co-expression genes in epilepsy. Genes were divided into various modules through hierarchical clustering, and different colors represent different modules. The gene that cannot be classified into any module by default is indicated in grey. **C** The heat map of epilepsy-related module–trait relationships. **D** Analysis of scale independence and mean connectivity based on the GSE58294 dataset. **E** Clustering dendrograms of co-expression genes in IS. **F** The heat map of IS-related module–trait relationships. IS: ischemic stroke; WGCNA: weighted gene co-expression network analysis.

To identify the disease-related genes, gene co-expression modules associated with epilepsy and IS were analyzed using the WGCNA of the DEGs. In this analysis, DEGs with similar expression patterns were assigned to the same module. Based on the GSE143272 epilepsy dataset, a power of 12 (scale-free R^2^ = 0.85) was chosen to ensure a scale-free network (Fig. 2A), and three modules were obtained, with each color representing each module (Fig. 2B). Subsequent correlation analysis revealed that two modules (MEblack and MEblue) were positively correlated with epilepsy (*r* > 0.8; *p* < 0.001) (Fig. 2C) and were considered the epilepsy-related key modules containing 594 DEGs. Similarly, based on the GSE58294 IS dataset, a power of 10 (scale-free R^2^ = 0.85) was selected to ensure a scale-free network (Fig. 2D), and seven modules were identified (Fig. 2E). The MEblack module was positively correlated with IS (*r* = 0.88, *p* = 2e − 14) (Fig. 2F) and was considered an IS-related key module. A total of 2623 DEGs were included in this module.

### Analysis of the shared genes of epilepsy and IS

Using Venn diagram analysis, 38 overlapping DEGs in the epilepsy- and IS-related key modules were obtained, which were considered as the shared genes of two diseases.

### Functional enrichment analysis for the shared genes of epilepsy and IS

**Fig. 3:**
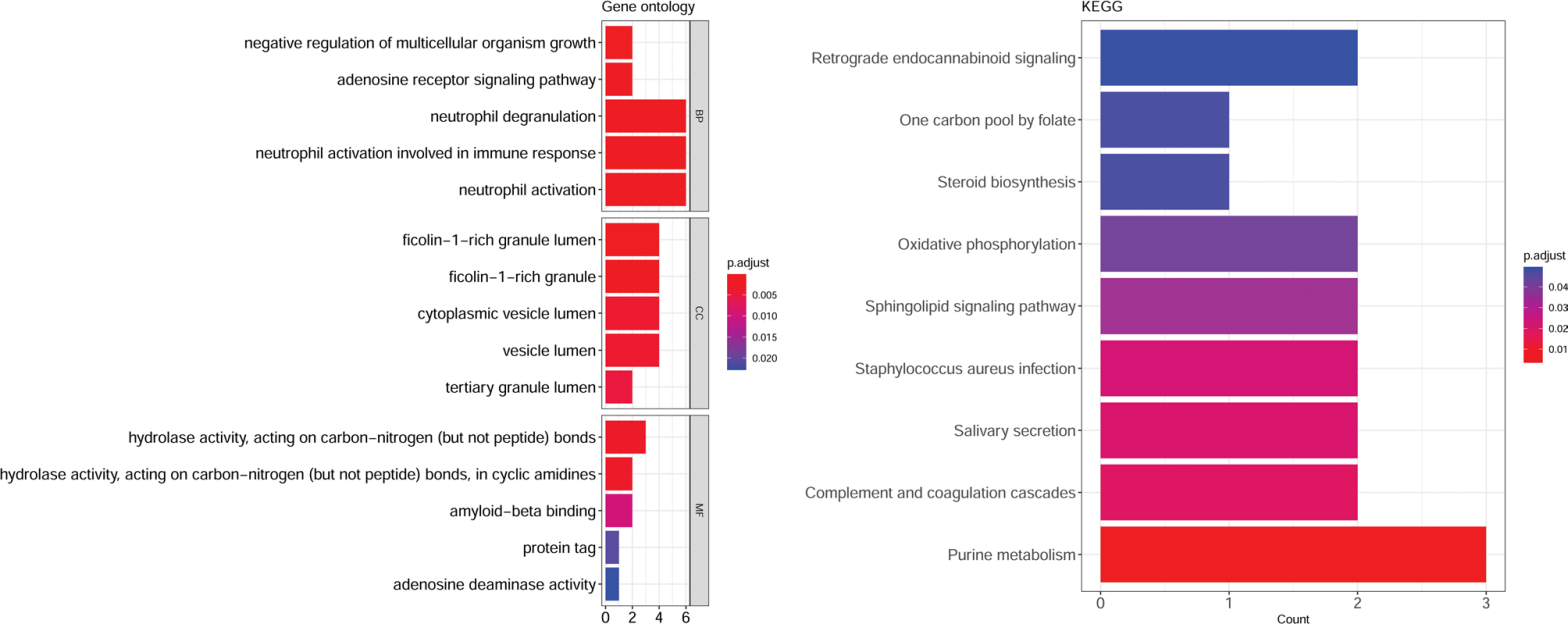
GO and KEGG pathway enrichment analysis for shared genes of epilepsy and IS. IS: ischemic stroke; GO: Gene Ontology; KEGG: Kyoto Encyclopedia of Genes and Genomes.

Functional enrichment analyses were performed to elucidate the potential functions of the shared genes in the two diseases. The shared genes were remarkably enriched in 189 GO BP terms such as neutrophil activation, 20 GO CC terms such as ficolin-1-rich granule lumen, 17 GO MF terms such as hydrolase activity, and 9 KEGG pathways such as purine metabolism (Fig. 3).

### PPI network analysis and hub gene identification

**Fig. 4:**
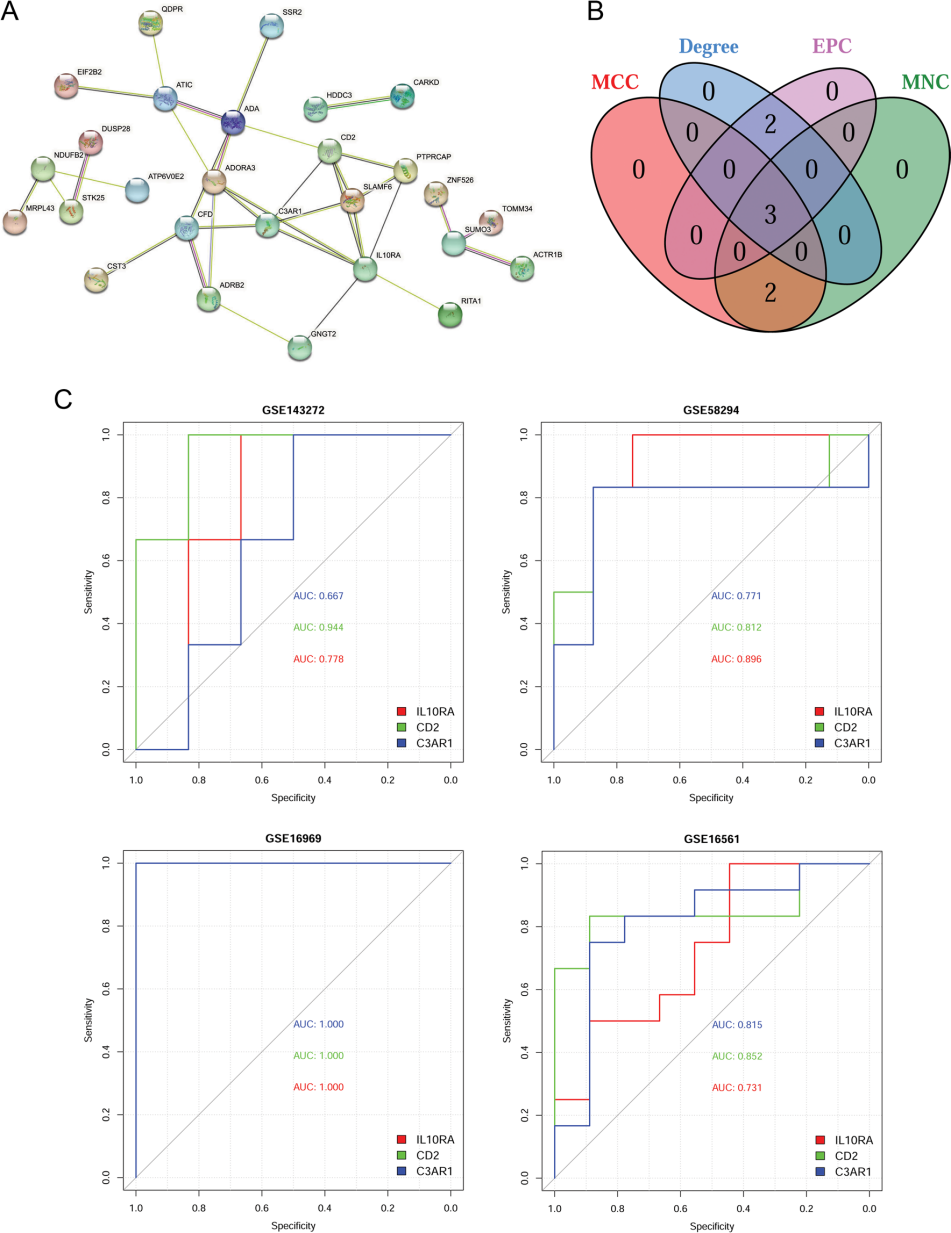
PPI network analysis and evaluating diagnostic efficacy of candidate hub-shared genes for epilepsy and IS. **A** PPI network constructed by the shared genes of two diseases. **B** Venn diagram shows the number of overlapping top genes in the PPI network based on four topological properties (MCC, MNC, degree, and EPC). **C** Evaluation of diagnostic efficacy of candidate hub-shared genes by ROC based on four datasets. IS: ischemic stroke; PPI: protein-protein interaction; MNC: maximal neighborhood component; MCC: maximal clique centrality; EPC: edge percolated component (EPC); ROC: receiver-operating characteristic; AUC: area under the curve.

Based on the information obtained from the STRING database, we constructed a PPI network including 26 shared genes of two diseases (Fig. 4A). The top five genes were respectively identified based on four topological analysis algorithms, including MNC, MCC, degree, and EPC. Intersection analysis of the top five genes from each topological analysis algorithm identified three overlapping genes as candidate hub genes for the two diseases (Fig. 4B), including interleukin 10 receptor subunit alpha (IL10RA), CD2 molecule (CD2), and complement C3a receptor 1 (C3AR1).

### Candidate hub-shared genes had high diagnostic efficacy for epilepsy and IS

The diagnostic efficacies of three candidate hub-shared genes, including L10RA, CD2, and C3AR1 were evaluated based on the gene expression data from all the datasets. The results showed that the AUC value of all these genes in training and validation datasets was > 0.66 (Fig. 4C). Therefore, all these genes had high diagnostic efficacies for epilepsy and IS.

### Validating the differential expression of the candidate hub-shared genes

**Fig. 5:**
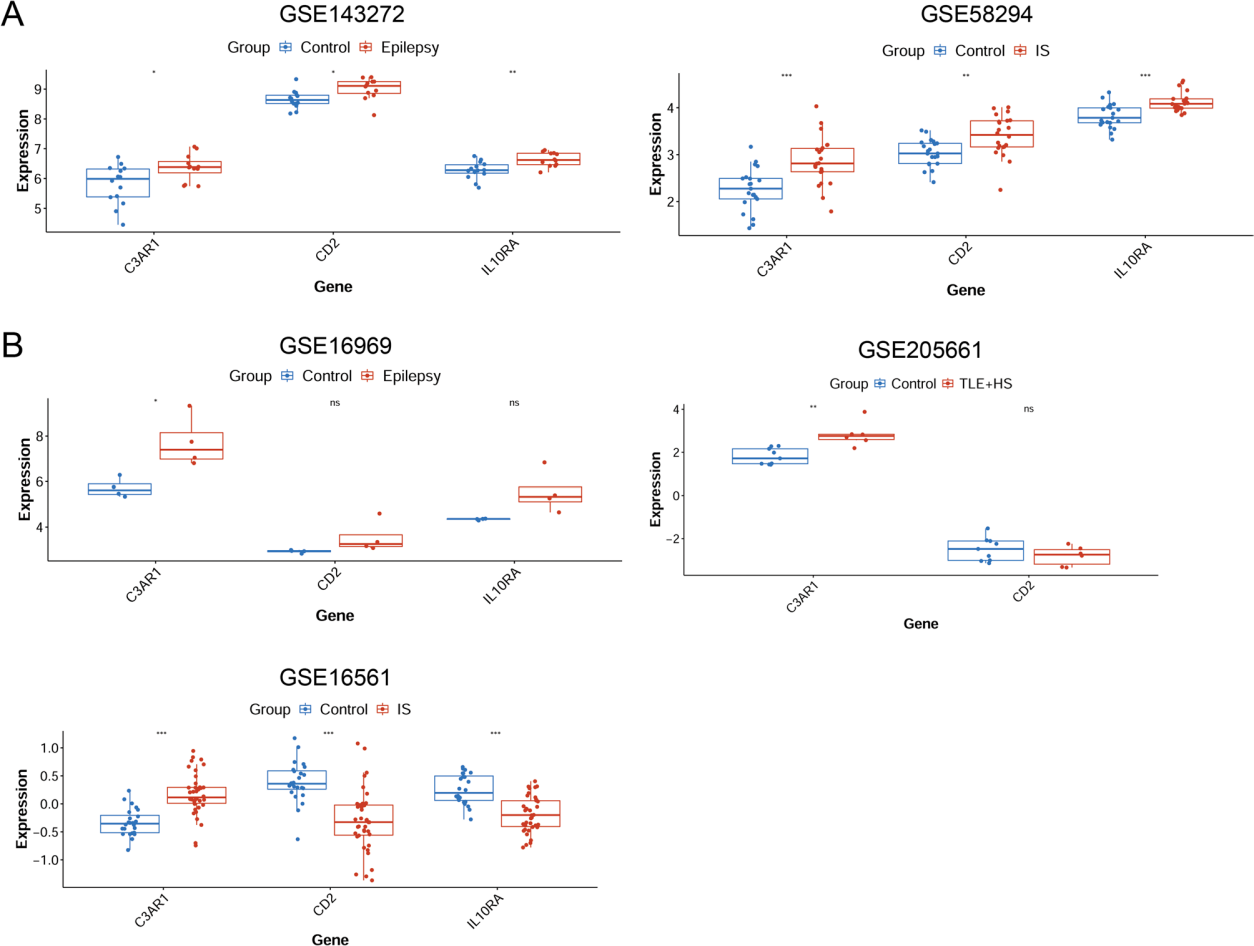
Validating the differential expression of the shared genes of epilepsy and IS. **A** Analysis of the expression of hub-shared genes using training datasets (the GSE143272 epilepsy dataset and GSE58294 IS dataset). **B** Analysis of the expression of hub-shared genes using validation datasets (the GSE16969 and GSE205661 epilepsy datasets and GSE16561 IS dataset). * *p* < 0.05, ** *p* < 0.01, and *** *p* < 0.001. IS: ischemic stroke; TLE + HS: temporal lobe epilepsy with hippocampal sclerosis.

We first extracted the expression of candidate hub-shared genes from the epilepsy and IS samples based on the training datasets of epilepsy (GSE143272) and IS (GSE58294). Compared to the respective control samples, the expression of IL10RA, CD2, and C3AR1 was upregulated in epilepsy and IS samples (Fig. 5A). Subsequently, we analyzed the differential expression of the candidate hub-shared genes based on the validation datasets of epilepsy (GSE16969 and GSE205661) and IS (GSE16561). The results showed that only C3AR1 expression was upregulated in the epilepsy and IS samples based on the validation datasets (Fig. 5B), which was similar to the results obtained from the training datasets. Thus, C3AR1 was recognized as a hub-shared gene in the two diseases.

###  Construction of the PPI network of C3AR1

**Fig. 6:**
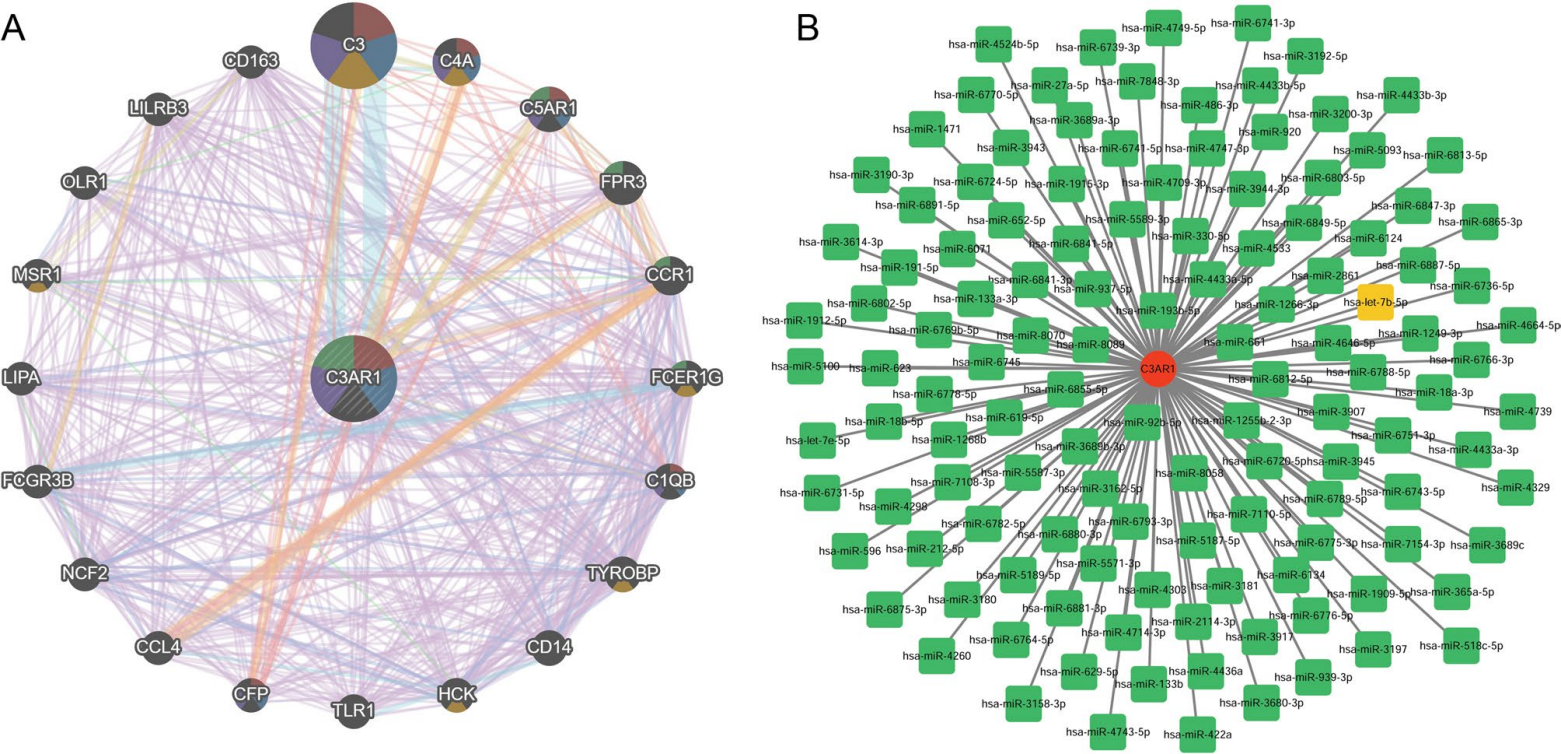
The PPI network of C3AR1 and upstream miRNA-C3AR1 network. **A** The PPI network of C3AR1 and its 20 cooperators was constructed using GeneMANIA. **B** The upstream miRNA-C3AR1 network. In this network, the red circle node is the hub-shared gene, the green square node is the miRNA, and the yellow square node is the common miRNA of the two diseases.

To explore the potential mechanisms of C3AR1 in the two diseases, a PPI network of C3AR1 was constructed using the GeneMANIA database (Fig. 6A). In this network, 20 cooperators of C3AR1, including the complement C3 (C3), complement C4A (C4A), and complement C5a receptor 1 (C5AR1) were identified.

### Analysis of the upstream MiRNAs of C3AR1

Using the HMDD, we identified 32 miRNAs associated with epilepsy and 40 miRNAs associated with IS. Subsequently, 10 common miRNAs associated with two diseases were identified. Moreover, 125 upstream miRNAs of C3AR1 were predicted using the miRWalk database, and an upstream miRNA-C3AR1 network was constructed (Fig. 6B). Notably, among the miRNAs upstream of C3AR1, only hsa-let-7b-5p was common in both diseases.

### Demographic data of clinical participants

This study included 34 participants: 14 children with epilepsy (7 girls and 7 boys) and 20 healthy controls (13 girls and 7 boys). No significant age differences were observed between the epilepsy and healthy control groups (mean age: 4.0 ± 1.5 years vs. 3.4 ± 0.8 years, *p* > 0.05), minimizing the impact of age as a confounding factor.

### Analysis of differential metabolites and pathway enrichment analysis

**Fig. 7:**
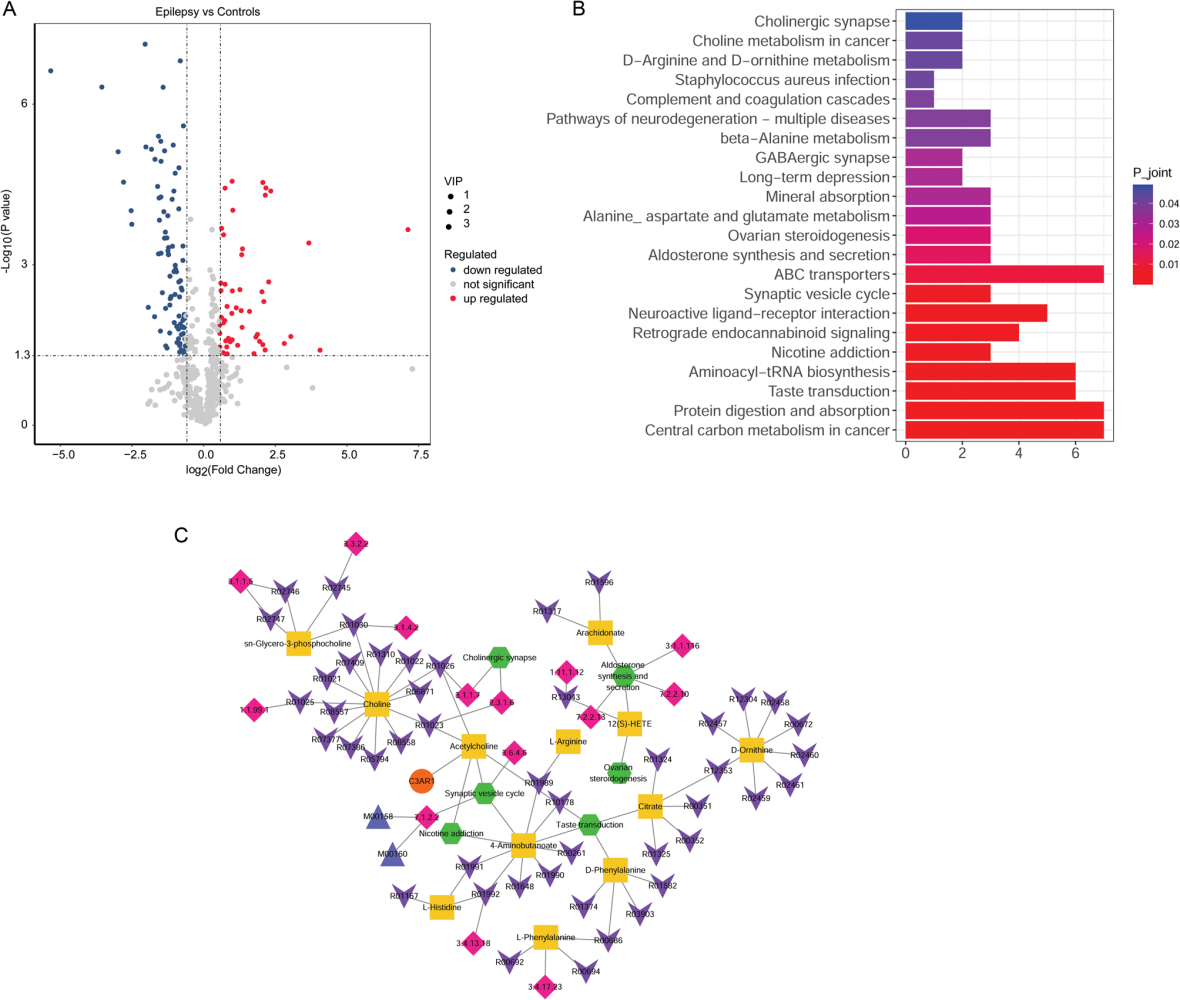
Identifying the differential metabolites, pathway enrichment analysis, and metabolic pathway network analysis. **A** Volcano plot of differential metabolites between the plasma samples from children with epilepsy and healthy controls. **B** Pathway enrichment analysis for differential metabolites. **C** Metabolic pathway network of C3AR1. In this network, the green hexagon node is the pathway, the blue triangle node is the module, the pink diamond node is the enzyme, the purple inverted triangle node is the reaction, the yellow square node is the differential metabolite, and the red circle node is the hub-shared gene.

Using nontargeted metabolomics analysis, 51 upregulated and 88 downregulated differential metabolites were identified between the plasma samples from children with epilepsy and healthy controls (Fig. 7A). Pathway enrichment analysis was performed to clarify the biological significance of C3AR1 and its metabolites. A total of 22 significantly enriched pathways containing 20 metabolites were identified (Fig. 7B).

### Metabolic pathway network analysis

We identified 22 significantly enriched pathways from the KEGG database. A metabolite pathway background network was constructed including 11,391 nodes, 33,332 edges, and 17 metabolites. The diffusion scores of the nodes were calculated using a diffusion algorithm. Subsequently, a metabolic pathway network was constructed using nodes with *p* < 0.05. The metabolic pathway network included 81 nodes, 85 edges, and 12 metabolites (Fig. 7C). Based on the diffusion algorithm, heat was believed to flow from the 12 differential metabolites and pass through the metabolite pathway background network. At rest, six pathways were identified: hsa04721, synaptic vesicle cycle; hsa04725, cholinergic synapses; hsa04742, taste transduction; hsa04913, ovarian steroidogenesis; hsa04925, aldosterone synthesis and secretion; and hsa05033, nicotine addiction. C3AR1 was involved in three pathways (hsa04721, hsa04742, and hsa05033) through interactions with the differential metabolite C01996 (acetylcholine). Acetylcholine was downregulated in the plasma samples from children with epilepsy.

## Discussion

IS has been identified as an important cause of epilepsy^38^. PSE is a common complication of IS that adversely affects the patients’ prognosis. Currently, the risk of PSE cannot be predicted adequately using clinical and radiological parameters^39^. Therefore, elucidating the common mechanisms underlying IS and epilepsy is necessary to uncover the underlying mechanisms and potential biomarkers of PSE. In this study, we investigated the shared genes of IS and epilepsy and explored their related mechanisms. The findings will improve our understanding of the common mechanisms underlying IS and epilepsy.

Using microarray technology, the expression data of thousands of genes for multiple diseases can be rapidly obtained, making it possible to elucidate the common pathogenesis of multiple diseases at the gene level^40,41^. WGCNA is a useful tool for discovering gene–gene and gene–disease relationships and has been widely applied to construct gene co-expression networks and identify key genes in disease-related networks^42–44^. In this study, we performed WGCNA and identified two epilepsy-related key modules containing 594 DEGs and an IS-related key module containing 2623 DEGs. PPI network analysis identified three candidate hub-shared genes from the 38 overlapping DEGs in the epilepsy- and IS-related key modules, including IL10RA, CD2, and C3AR1. These data suggest that these shared genes may be involved in the development of IS and epilepsy.

IL-10 is a multifunctional anti-inflammatory cytokine that plays a neuroprotective role in the brain^45,46^. It can inhibit IL-1β production and suppress inflammasome activation of microglia in epileptic seizures^47^. IL-1β can also predict seizure recurrence after the first epileptic seizure among IS patients and may serve as a promising prognostic biomarker for PSE^48,49^. Moreover, IL-10 is positively associated with the risk of stroke^50,51^. IL-10 activates downstream signaling by binding to the IL-10 receptors (IL-10RA and IL10RB)^52^. Polymorphisms in IL10, IL10RA, and IL10RB genes are associated with IS in terms of hypertension^53^. CD2 belongs to the immunoglobulin superfamily and plays a key role in mediating T and natural killer (NK) cell activation^54^. Cluster of differentiation 8 (CD8+), cluster of differentiation 4 (CD4+), and NK T cells are recruited within 24 h of IS and play a crucial role in regulating the inflammatory response after injury^55^. T-cell numbers are associated with neuronal loss in medial temporal lobe epilepsy^56^. These findings suggest that CD2 contributes to epilepsy and IS by affecting T and NK cell activation. C3AR1 is a key regulator of neuronal tau pathogenesis and has been implicated in the immune network of the CNS^57^. Tau protein hyperphosphorylation has been observed in animal models of epilepsy^58^ and in patients with epilepsy^59^. Tau protein also contributes to brain damage following stroke as seen in an animal model of stroke^60^ and has been suggested as a potential therapeutic target for IS^61^. Moreover, Tau protein is reported to have a sensitivity of 100% and a specificity of 73% for predicting PSE^39^. These data suggest that C3AR1 contributes to epilepsy and IS by regulating the pathogenesis of the neuronal tau protein. In our study, ROC analysis revealed that the diagnostic efficacy of L10RA, CD2, and C3AR1 was high, with AUC values > 0.66 in the training and validation datasets. Therefore, these genes may serve as promising biomarkers for the diagnosis of IS and epilepsy. However, differential expression validation showed that only the expression trend of C3AR1 was consistent in both the training and validation datasets. The differential expression and diagnostic value of L10RA, CD2, and C3AR1 need to be validated using additional datasets or clinical cohorts.

miRNAs are small noncoding RNA molecules of 22 nucleotides in length. Accumulating evidence has confirmed that miRNAs are key regulators of IS^62^, epilepsy^63^, and PSE^64^. In this study, we found that hsa-let-7b-5p is a common miRNA in epilepsy and IS and can target C3AR1. A previous study has shown that knockdown of let-7b-5p reverses the effect of repetitive transcranial magnetic stimulation on microglia phenotype, and play a key role in neurological recovery and preventing ischemic stroke^65^. Combined analyses of Fas and hsa-let-7b-5p expression have revealed them to be promising biomarkers for predicting poor neurological outcomes in patients with IS patients^66^. Although the role of hsa-let-7b-5p in epilepsy has not been reported, our results imply that hsa-let-7b-5p-C3AR1 axis may be a potential common regulatory mechanism mediating IS and epilepsy.

Several metabolites like lactate, glutamate, and citrate as well as metabolic pathways such as glycine, serine, and threonine metabolism were recently shown to be involved in epilepsy, providing a novel perspective for exploring promising biomarkers and therapeutic targets for epilepsy^67^. Thus, we conducted a nontargeted metabolomics analysis to identify differential metabolites in the plasma samples of children with epilepsy and subsequently analyzed the key metabolites that may be regulated by C3AR1. We found that acetylcholine was downregulated in the plasma samples of children with epilepsy and that C3AR1 was involved in three pathways (hsa04721, synaptic vesicle cycle; hsa04742, taste transduction; and hsa05033, nicotine addiction) through interaction with acetylcholine. Acetylcholine is the main stimulant of the autonomic nervous system and plays a key role in signal transmission via the cholinergic and nicotinic receptors. Increasing evidence has highlighted the role of acetylcholine and cholinergic neurotransmission in the pathogenesis of epilepsy^68–70^. Moreover, the synaptic vesicle cycle controls neurotransmitter release and is implicated in epilepsy progression^71^. Nicotine addiction is also an important pathway that mediates the role of drugs in treating epilepsy^72^, suggesting a potential role of nicotine addiction in epilepsy. Overall, our data suggest that C3AR1 may contribute to PSE development by regulating acetylcholine and related pathways such as the synaptic vesicle cycle and nicotine addiction.

Several limitations should be considered when interpreting the findings of this study. Firstly, the hub-shared genes such as C3AR1 and its related miRNAs like hsa-let-7b-5p were obtained from bioinformatics analyses of publicly available datasets, and their expression was not validated in clinical samples. Validating their expression and investigating how C3AR1 interacts with other relevant molecules and its specific mechanisms in disease pathology is crucial for understanding its potential as a diagnostic biomarker. Secondly, the functions of hub-shared genes, especially C3AR1 were not explored in depth. Further functional studies, such as gene knockout or overexpression experiments, are required to investigate the role of in epilepsy and ischemic stroke. Thirdly, fourteen plasma samples from children with epilepsy were used in this study. The sample size was small, which may reduce the statistical power. Lastly, this study only validated changes in metabolites in plasma samples from epilepsy patients, without assessing metabolite alterations in IS. Future studies should be conducted to investigate metabolic changes in IS patients as well to help clarify the similarities and differences between epilepsy and IS at the metabolic level, further supporting the potential of shared genes as biomarkers for both conditions. Furthermore, given the complexity of these diseases, future research may focus on identifying a panel of biomarkers rather than relying on a single gene.

## Conclusions

Our findings reveal that the key shared genes, especially C3AR1, may be implicated in the development of IS and epilepsy and could serve as potential diagnostic biomarkers for both diseases. Furthermore, C3AR1 may contribute to PSE development by interacting with hsa-let-7b-5p and acetylcholine. These findings may aid in the precise diagnosis and treatment of IS and epilepsy.

## Supplementary Information

Below is the link to the electronic supplementary material.

Supplementary Material 1

Supplementary Material 2

Supplementary Material 3

## Data Availability

Data is provided within the manuscript or supplementary information file. Further enquiries can be directed to the corresponding author.

## Abbreviations

IS: Ischemic stroke
IL10RA: Interleukin 10 Receptor Subunit Alpha
CD2: Cluster of differentiation 2
C3AR1: Complement component 3a receptor 1
GEO: Gene Expression Omnibus
WGCNA: Weighted gene co-expression network analysis
PPI: Protein–protein interaction
ROC: Receiver-operating characteristic
DEG: Differentially expressed genes
PSE: Post-stroke epilepsy
DAMP: Damage-associated molecular patterns
CNS: Central nervous system
GO: Gene Ontology
KEGG: Kyoto Encyclopedia of Genes and Genomes
MF: Molecular function
CC: Cellular component
BP: Biological processes
MNC: Maximal neighborhood component
MCC: Maximal clique centrality
EPC: Edge-percolated component
AUC: Area under the curve
HMDD: Human microRNA Disease Database
NK: Natural killer
CD8: Cluster of differentiation 8
CD4: Cluster of differentiation 4
CD2: Cluster of differentiation 2
