# Supplementary figures and images for: Identifying the shared genes and their related microRNAs, metabolites, and pathways in ischemic stroke and epilepsy
Source: Sci Rep. 2026 Feb 10;16:8166. doi: 10.1038/s41598-026-39299-5 (PMC12963536; doi:10.1038/s41598-026-39299-5)

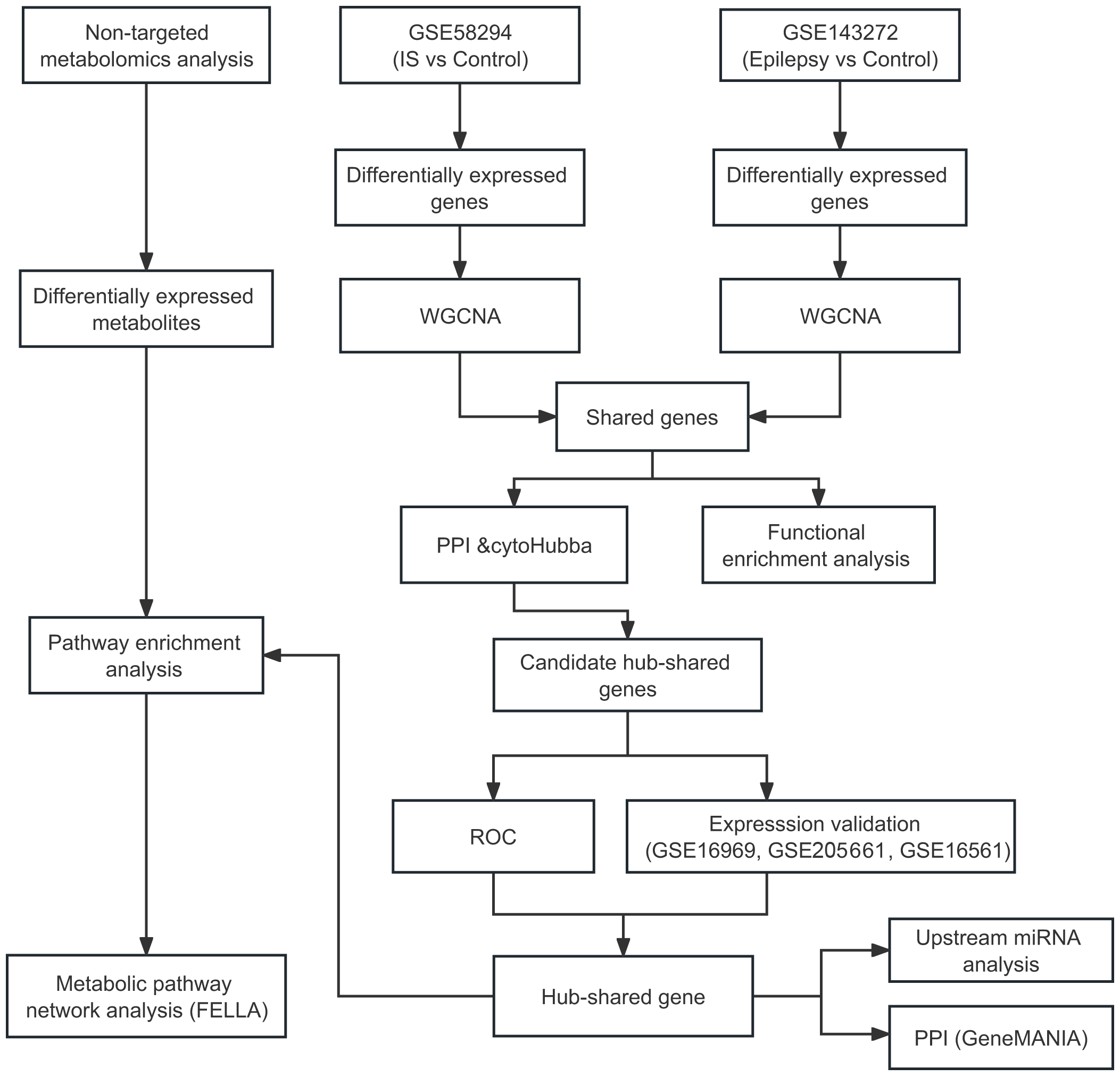

Supplement: Supplementary file 1 — Supplementary Material 1 [file 41598_2026_39299_MOESM1_ESM.tif]
